# Electric Field Effects on Curved Graphene Quantum Dots

**DOI:** 10.3390/mi14112035

**Published:** 2023-10-31

**Authors:** Sergio de-la-Huerta-Sainz, Angel Ballesteros, Nicolás A. Cordero

**Affiliations:** 1 Physics Department, Universidad de Burgos, 09001 Burgos, Spain; shuerta@ubu.es (S.d.-l.-H.-S.); angelb@ubu.es (A.B.); 2 International Research Center in Critical Raw Materials for Advanced Industrial Technologies (ICCRAM), Unversidad de Burgos, 09001 Burgos, Spain; 3 Institute Carlos I for Theoretical and Computational Physics (IC1), 18016 Granada, Spain

**Keywords:** graphene, nanoflake, electric field, quantum revival, DFT

## Abstract

The recent and continuous research on graphene-based systems has opened their usage to a wide range of applications due to their exotic properties. In this paper, we have studied the effects of an electric field on curved graphene nanoflakes, employing the Density Functional Theory. Both mechanical and electronic analyses of the system have been made through its curvature energy, dipolar moment, and quantum regeneration times, with the intensity and direction of a perpendicular electric field and flake curvature as parameters. A stabilisation of non-planar geometries has been observed, as well as opposite behaviours for both classical and revival times with respect to the direction of the external field. Our results show that it is possible to modify regeneration times using curvature and electric fields at the same time. This fine control in regeneration times could allow for the study of new phenomena on graphene.

## 1. Introduction

There is no doubt that there has been a long-lasting wave of advancements and innovation revolving around graphene, with strange phenomena and potential applications appearing on a yearly basis. Almost every field seems to have an application where the extraordinary properties of graphene (either pristine or oxidised) can shine, from engineering [1,2,3,4] to catalysis [5,6,7,8], medicine [9,10,11,12,13], sensing [14,15,16], optics/electromagnetics [17,18,19,20], hydraulics [21,22], and energy management [23,24,25], to cite a few examples.

In spite of the mechanical properties of graphene being quite remarkable, the electronic ones are by far the main factor responsible for its attractiveness as a research topic and the basis for several groundbreaking applications. The peculiar band structure of graphene, calculated by Wallace many decades ago [26] by an approximated yet still useful method, presents the electrons as massless Dirac quasiparticles [27], allowing them to reach high speeds [28] that result in its incredible electrical conductivity [29,30]. This, alongside the ballistic transport [31,32] observed on non-perturbed systems, makes graphene a great replacement for common conductive materials. Also, due to its near-relativistic behaviour, graphene is often used as an experimental analogue in order to replicate otherwise nearly impossible to measure phenomena, like the Klein paradox [33,34,35], Zitterbewegung [36,37,38,39], or the properties of spacetime with a negative curvature [40,41,42,43].

Perhaps one of its most interesting applications derives from the interaction between graphene and an external electric field. As the band structure is so important for its electronic properties, it is quickly deduced than they can be modified and tuned easily by adjusting the field, allowing feats such as moving the Fermi energy between bands and changing the type and density of charge carriers—the ambipolar electric field effect [44,45]—or widening the gap between valence and conduction bands to obtain an insulating behaviour, if needed. Taking also into account the high mobility of electrons in this material and its nanometric scale, graphene is a very promising alternative material for fast and incredibly small electronic components, such as field-effect transistors [46]; the ballistic transport would ensure a quick response and reduced heating, and the robustness and transparency of the material could extend its use to flexible devices and screens [47,48,49].

A known effect of an electric field on graphene is the appearance of the curvature. When a transversal field is applied, the material bends alongside the direction of the field, as has been both predicted and observed recently [50,51,52,53]. While curved graphene is not a strange view, as it naturally appears in the material itself even when suspended and unperturbed [29,54,55,56,57,58], the effects of the deformation on the electronic behaviour are notable. Intrinsic ripples are known to disturb the otherwise free electron paths [27], and local deformations, known as nanobubbles, can be easily produced in the material, allowing for a localised tuning of electric conductivity and more exotic phenomena, like pseudo-magnetic field generation [59,60]. It has been recently shown that double quantum dots can be created by these pseudo-magnetic fields in nanobubbles and that their quantum states can be manipulated to create a controllable qubit [61].

Thus, our aim in this paper about graphene nanoflakes is to study the effects of an electric field on a spherically deformed system, analysing the electronic spectrum via temporal evolution through the quantum regeneration phenomenon—the partial or total recovery of the initial state of a wavepacket after a certain amount of time, known as the regeneration or revival time. An atomistic method, like the Density Functional Theory (DFT), will serve as the main tool, as it has previously for nanoflakes with spherical geometries [62], as well as those with cylindrical and hyperbolical geometries [63], in the absence of an external field. Some of those results will be used here for comparative purposes.

## 2. Materials and Methods

As this work is a continuation of our previous research about quantum revivals in graphene, the methodology, as well as the system, will remain the same: a hexagonal graphene nanoflake of 10 benzenic rings per edge, with zig-zag edges and hydrogen passivated; the shape will be achieved through the application of the spherical surface equation, Equation (1), for several values of *R* between 30 Å and 1000 Å:

(1) $$z=\sqrt{R^{2}-x^{2}-y^{2}}\;.$$

This flake will be subjected to a structural optimisation calculated within the Local Density Approximation (LDA) using the Gaussian 16 package [64] until forces on all atoms are below 0.0045 Hartree/Bohr and displacements below 0.0018 Bohr. While other common functionals, such as B3LYP or Generalised Gradient Approximations (GGAs), can be used for smaller systems, LDA results for graphene systems are of a similar or higher quality [65,66], they require lower computational work, and this functional has been successfully used to study carbon nanostructures [67,68,69,70,71,72,73]. Meta-GGAs provide better accuracy than LDA but the computing time is much higher. Taking into account that some of our calculations needed 1 year of CPU time at the LDA level, using those functionals was out of the question. Therefore, we followed the advice in a recent benchmark paper on ab initio descriptions of carbon nanomaterials using 15 different functionals [74] and used LDA. The basis set will remain 6-31G** (with p and d functions as polarisation aids) [75], as it has provided good quality results on all calculations made so far.

**Figure 1:**
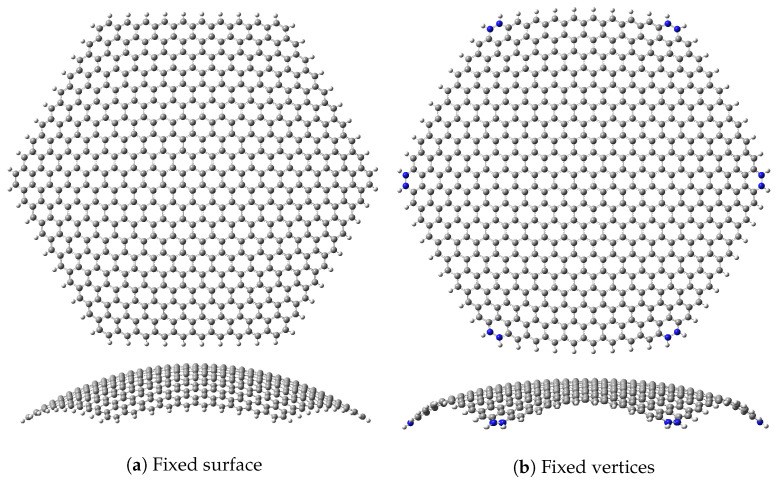
Top (upper row) and side (lower row) views of the two boundary conditions for a spherical nanoflake with initial R=40Å. Clear differences between both optimised structures can be seen, especially on the edges. The atoms fixed in the second case are highlighted in blue. Images generated with Gaussview 6 [82].

Highly spherical nanobubbles can be achieved with external electric fields [51], so a perfect spherical deformation could be enough for this study; however, for the sake of experimental realism and fair comparisons, two different sets of geometric restrictions will be applied to the nanoflake (Figure 1): (i) maximal restriction, forcing all carbon atoms to lie on the spherical surface and (ii) minimal restriction, forcing only the 12 carbon atoms on the vertices to remain on the spherical surface. The first case may seem purely academic, but it is a first approximation to a possible experimental setup. One method to stabilise graphene for electronic applications is using hexagonal boron nitride (h-BN) as a substrate [76,77,78]. A spherical boron nitride fullerene [79,80,81] could be used to support a spherical graphene nanoflake.

Regarding the electric field, considering the original flat nanoflake was constructed in the *xy* plane, the fields applied will be in the direction of the *z* axis—and thus perpendicular to the unperturbed flake plane—with three intensities (0.0050 a.u., 0.0100 a.u., and 0.0250 a.u.) in two opposite directions (one in the same direction as the flake deformation and the other in the opposite direction) each, labelled as ±50, ±100, and ±250, with the positive sign corresponding to the field in the same direction as the flake deformation. These fields could seem to have an astonishing intensity, considering that a field of 1 atomic unit equals 5 × $10^{11}\;Vm^{-1}$, but at the nanometric scale such fields can be achieved by setting the electrodes close enough.

## 3. Results

The study of the effect of an electric field has been carried out from both mechanical and electronic points of view, the first through the curvature energy and the latter by calculating the temporal evolution of a wavepacket and checking if quantum regeneration occurs. To this end, spherical surfaces of various curvature radii have been used to deform the flake, performing an optimisation on each combination of radius, restriction set, and electric field.

### 3.1. Curvature Energy

As was the case in our previous works, the mechanical study will use the total energy of each nanoflake, taking the flat configuration as the origin of energies, i.e.,

(2) $$E=E_{\mathrm{curved}}-E_{\mathrm{flat}}\;,$$

where $E_{\mathrm{curved}}$ is the energy of the curved flake and $E_{\mathrm{flat}}$ is the energy of the planar configuration (both energies calculated with the same electric field).

**Figure 2:**
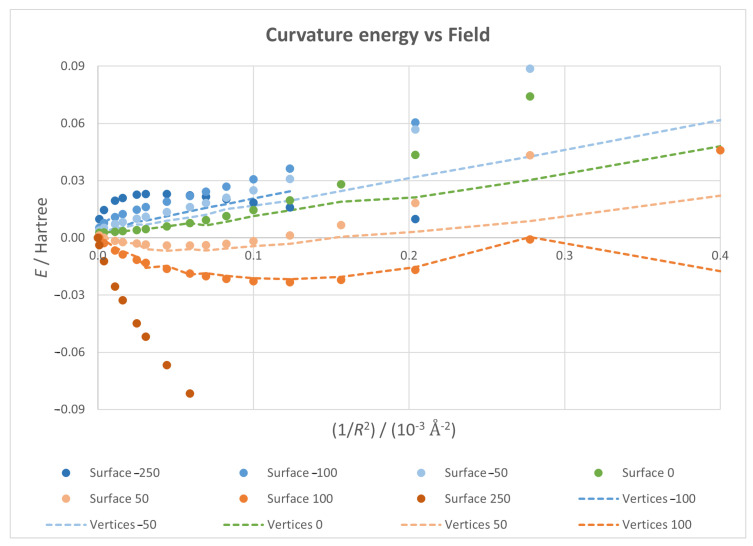
Curvature energy vs. $1/R^{2}$ plots for different values of field intensity and direction—with positive fields in red tones and negative ones in blue tones—with markers and dotted lines for total and minimal restriction, respectively.

Figure 2 shows the curvature energy plotted against $1/R^{2}$—which is the Gaussian curvature of the flake for the maximal restriction case—for different field strengths. In order to make figures easier to understand, we have used point markers for results corresponding to maximal restriction and dashed lines for results corresponding to minimal restriction. In this latter case, the lines are merely guides for the eye connecting the results corresponding to consecutive values of $1/R^{2}$ (we have used the same set of values as for the maximal restriction case). While all curves show that, as expected, flakes with higher curvatures have higher energies, the trends vary depending of the relative direction of the field; for zero fields, the plot has a nearly parabolic shape, with the flat case being the lowest energy geometry; for positive fields, the plots are also parabolic, but the minimal energy is reached for a non-planar structure, which showcases the curvature induced on the material as a transversal field is applied; lastly, negative fields result in an initial rise in energy at lower curvatures, as the field is trying to induce a deformation opposite to the pre-existing one, an effect countered by bending as curvature grows.

Stronger fields lead to analogous behaviours, but the effects are bigger as the field increases. The optimal structure is more curved and less energetic for positive fields, while the initial energy increase appears earlier and is greater for negative fields. However, a strong enough negative field can override the curvature effect and produce a maximum in energy.

### 3.2. Electric Dipole Moment

Due to its close interaction with external fields, the behaviour of the electric dipole moment has also been studied. From the flake shape and symmetry, a null moment would be expected for the flat case and a non-zero moment on the negative *z* axis for the spherical ones; this moment would be intensified with positive fields, as the charge distribution that originated it becomes more pronounced and decreases or even flips with negative fields.

**Figure 3:**
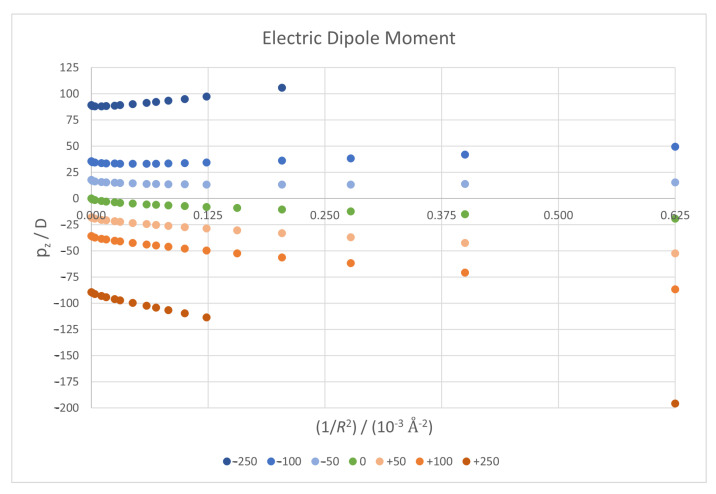
*z* component value of the electric dipole moment vector against $1/R^{2}$ for all seven field cases.

In Figure 3, we have plotted the *z* component of the dipole moment against $1/R^{2}$ for each of the seven field values. As expected, in general, the dipole along the *z* direction grows in absolute magnitude with an increasing curvature, since a bigger curvature allows for a greater charge displacement. In the absence of an external electric field (green points), the planar structure ($1/R^{2}=0$) has no global dipole moment due to the symmetry of the electron cloud with respect to the graphene plane.

**Figure 4:**
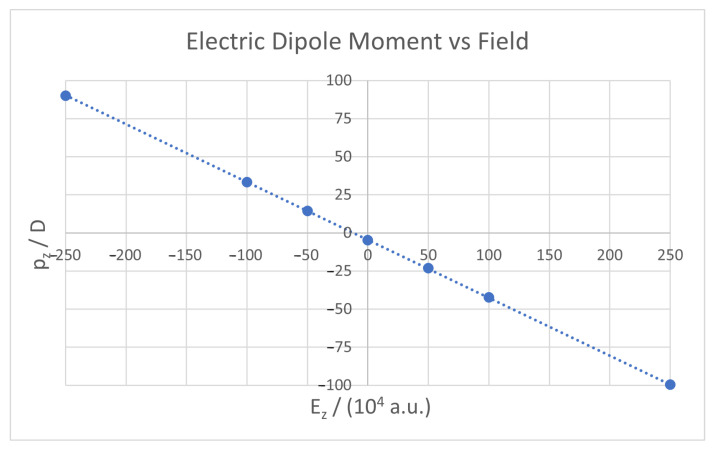
*z* component of the electric dipole against external field, for a nanoflake with *R* = 150 Å. Because of the relatively small variation within the same field, only a single case is displayed here, for clarity.

Figure 4 shows the dependence of one particular case with the field, showing an almost perfectly linear trend, as expected. Without an electric field, the dipole moment is not exactly zero because curvature causes the centre of charge of the electron cloud (negative) to not be at the exact same position as the centre of charge of the atomic nuclei (positive). The first is slightly below the second and this fact creates a very small downwards dipole. With a positive electric field, i.e., the field pointing upwards, atomic nuclei tend to move up (and with them the centre of positive charge) while the electronic cloud tends to move down (and with it the centre of negative charge). This translates into a larger negative dipole moment that increases with the strength of the field. The opposite happens when the applied electric field is negative, i.e., when the field points downwards, atomic nuclei tend to move down, while the electronic cloud tends to move up. This results in a larger positive dipole moment that increases with the field strength.

It is interesting to see the behaviour for negative fields in Figure 3, where in this case the dipole component initially decreases before growing. For positive fields, the charge shift induced by the field and the curvature work together to achieve a greater downwards dipole; for negative fields, they oppose, and as charges can move longer distances in these conditions—the centre and edges are farther away—the curvature achieves a totally opposite effect.

The direction of the dipolar moment suggests a higher electron density on the sphere inner region, something already studied on larger curved carbon nanostructures [30,83], in which the curvature reduces the effective distance between atoms and provokes a rehybridisation on them, with a higher overlap and a consequent energy decrease. While there are works studying the connection between the local charge distribution and graphene rippling [84], these are oriented to a more continuous and extended system; alas, it is not justified to assume a direct connection between our small, static system and a much bigger and more dynamic phenomenon like graphene corrugation.

### 3.3. Regeneration Times

Quantum regeneration phenomena will be studied using the electronic spectrum of each nanoflake as a starting point. For that purpose, we define a wavepacket as a linear combination of eigenfunctions $u_{n}$ with varying coefficients $c_{n}$:

(3) $$|\Psi (0)\rangle =\sum_{n=0}^{\infty }c_{n}|u_{n}\rangle \;.$$

We can calculate the state of the packet after an arbitrary time *t* in order to study its time evolution:

(4) $$|\Psi (t)\rangle =\sum_{n=0}^{\infty }c_{n}|u_{n}\rangle e^{-\frac{i}{\hbar }E_{n}t}\;,$$

with $E_{n}$ being the eigenvalue corresponding to the $|u_{n}\rangle$ eigenstate. The weights of each eigenfunction will follow a Gaussian distribution defined by level numbers—positive integers for unoccupied states and negative for occupied ones—centred around the LUMO+4 ($n_{0}$ = 5) and a width σ of 0.7, ensuring a wavepacket narrow enough to observe quantum regeneration:

(5) $$c_{n}=\frac{1}{\sigma \sqrt{\pi }}e^{\frac{-{\left(n-n_{0}\right)}^{2}}{2\sigma^{2}}}\;.$$

Using level numbers allows us to use an analytical approximation for the regeneration times, as described in [85]. This approximation begins with a Taylor expansion of the spectrum E(n) around the central level $n_{0}$:

(6) $$E_{n}=E_{n_{0}}+E_{n_{0}}^{\prime }\left(n-n_{0}\right)+\frac{1}{2!}E_{n_{0}}^{\prime \prime }{\left(n-n_{0}\right)}^{2}+\frac{1}{3!}E_{n_{0}}^{\prime \prime \prime }{\left(n-n_{0}\right)}^{3}+\ldots \;.$$

By combining Equations (4) and (6), we reach an expanded evolved state:

(7) $$|\Psi (t)\rangle =\sum_{n=0}^{\infty }c_{n}|u_{n}\rangle e^{-\frac{i}{\hbar }\left(E_{n_{0}}+E_{n_{0}}^{\prime }\left(n-n_{0}\right)+\frac{1}{2!}E_{n_{0}}^{\prime \prime }{\left(n-n_{0}\right)}^{2}+\frac{1}{3!}E_{n_{0}}^{\prime \prime \prime }{\left(n-n_{0}\right)}^{3}+\ldots \right)t}\;,$$

in which each term on the exponential function constitutes a temporal scale. This expression allows for the definition of several regeneration times, directly connected to the spectrum derivatives:

(8) $$T_{\mathrm{Zb}}=\frac{\pi \hbar }{|E_{n_{0}}|}\;,$$

(9) $$T_{\mathrm{Cl}}=\frac{2\pi \hbar }{|E_{n_{0}}^{\prime }|}\;,$$

(10) TRe=2πħ|En0″|/2!,and

(11) $$T_{\mathrm{Sup}}=\frac{2\pi \hbar }{|E_{n_{0}}^{\prime \prime \prime }|/3!}\;.$$

The main times of interest here will be $T_{\mathrm{Cl}}$, the classical time, and TRe, the revival time, as they will be easier to observe and rationalise. Being dependent on the first and second derivatives, respectively, the classical time gives information about the absolute value of the energy differences and thus the compression of the spectrum, while the revival time informs about the similarity between energy differences and thus about the regularity of the spectrum.

$T_{\mathrm{Zb}}$ is related to Zitterbewegung, a relativistic phenomenon only observed on particular wavepackets (cat states), different from ours. Here, it is just a mere phase factor. In contrast, $T_{\mathrm{Sup}}$, the super-revival time, would correspond to a higher scale oscillation on the temporal evolution, which is difficult to detect due to the decreasing value of higher-order derivatives and as such will not be considered either.

The interpolating function selected to calculate the derivatives appearing in these times is a second-degree polynomial, obtained by a least squares fit to the three central levels of the wavepacket. By using three points—the level numbers and their energies—an exact expression for the polynomial can be obtained:

(12) $$E\left(n\right)=\frac{b-a}{2}\left(n^{2}-n_{0}^{2}\right)+\frac{1}{2}\left(a+b+2an_{0}-2bn_{0}\right)n+E_{n_{0}}\;,$$

with $n_{0}$ being the central orbital number, $E_{n_{0}}$ its energy, and *a* and *b* the absolute energy differences with the lower ($n_{0}-1$) and upper ($n_{0}+1$) levels, respectively. Both first and second derivatives can be analytically computed easily:

(13) $$E^{\prime }\left(n\right)=\left(b-a\right)n+\frac{1}{2}\left(a+b+2an_{0}-2bn_{0}\right)$$

and

(14) $$E^{\prime \prime }\left(n\right)=b-a\;.$$

These expressions will be useful in later sections.

As for the actual temporal evolution, it can be monitored through the autocorrelation function, defined as the overlap of initial (t=0) and later (t>0) states:

(15) $$A\left(t\right)=\langle \Psi (0)|\Psi (t)\rangle =\sum_{m=0}^{\infty }\sum_{n=0}^{\infty }c_{m}^{*}c_{n}\langle u_{m}|u_{n}\rangle e^{-\frac{i}{\hbar }E_{n}t}$$

Working with orthonormal states in the wavepacket makes it possible to substitute Kronecker’s delta functions for the overlap integrals and cancel all cases where n≠m:

(16) $$A\left(t\right)=\sum_{m=0}^{\infty }\sum_{n=0}^{\infty }c_{m}^{*}c_{n}e^{-\frac{i}{\hbar }E_{n}t}\delta_{mn}=\sum_{n=0}^{\infty }|c_{n}{|}^{2}e^{-\frac{i}{\hbar }E_{n}t}\;.$$

The physically sound quantity is the squared modulus of the autocorrelation function:

(17) $${\left|A\left(t\right)\right|}^{2}=\sum_{m=0}^{\infty }\sum_{n=0}^{\infty }|c_{m}{|}^{2}|c_{n}{|}^{2}e^{-\frac{i}{\hbar }\left(E_{n}-E_{m}\right)t}\;.$$

Taking into account the definition of the cosine function, we can rewrite this expression as follows:

(18) $${\left|A\left(t\right)\right|}^{2}=\sum_{n=0}^{\infty }{\left|c_{n}\right|}^{4}+\sum_{n=0}^{\infty }\sum_{m<n}^{\infty }2|c_{m}{|}^{2}|c_{n}{|}^{2}\cos\left(\omega_{mn}t\right)\;,$$

where $\omega_{mn}=\left(E_{m}-E_{n}\right)/\hbar$. This formula relates the temporal evolution to the energy differences between different levels on the packet, rather than the energies themselves. As a sum of cosines, a complete revival is guaranteed (if the spectrum is commensurable) after a long enough time, when the least common multiple of all individual periods is reached.

We can focus on our particular wavepacket to further simplify this expression. While is it true that the wavepacket comprises five levels, the coefficient distribution and their squares on the general formula makes the three central ones (4, 5, and 6) the main contributors, so the general expression can be simplified. We can ignore any terms involving other levels. We can go even further and remove also the $\omega_{46}$ term, as the distribution is narrow enough to make its contribution barely noticeable, resulting in the following expression:

(19) $${\left|A\left(t\right)\right|}^{2}\approx 2|c_{4}{|}^{2}|c_{5}{|}^{2}\cos\left(\frac{a}{\hbar }t\right)+2|c_{5}{|}^{2}{\left|c_{6}\right|}^{2}\cos\left(\frac{b}{\hbar }t\right)\;,$$

where we have removed the first sumatory and proportionality constants, as the periodicity of the function remains the same, and *a* and *b* are, like before, the energy differences between level 5 and levels 4 and 6, respectively. Being a sum of cosines, and considering $c_{4}$ and $c_{6}$ are identical due to the distribution’s symmetry, it can be easily converted to a product of cosines:

(20) $${\left|A\left(t\right)\right|}^{2}\approx 4|c_{4}{|}^{2}{\left|c_{5}\right|}^{2}\cos\left(\frac{a+b}{2\hbar }t\right)\cos\left(\frac{a-b}{2\hbar }t\right)\;.$$

The two new arguments found here are interesting: the first one is the average of both main energy differences, and the second gives information about how similar they are, which makes them perfect candidates for the origin of both classical and revival times, respectively.

**Figure 5:**
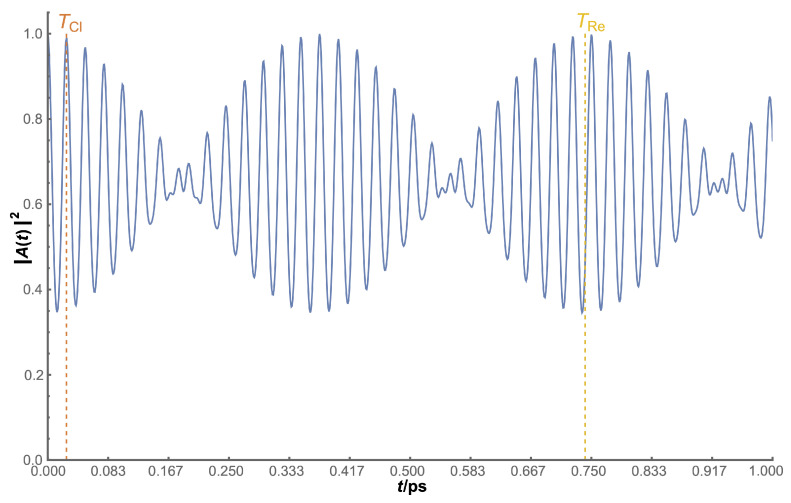
Temporal evolution plot for a fixed surface nanoflake, with *R* = 150 Å in a −50 field, as an exemplary case. Dotted lines represent the analytical values calculated for both $T_{\mathrm{Cl}}$ and TRe.

Finally, Figure 5 shows a typical view of ${\left|A\left(t\right)\right|}^{2}$ plotted against time, with a clear pattern of a high-frequency oscillation modulated by a low-frequency one, whose periods correspond to the values of $T_{\mathrm{Cl}}$ and TRe, respectively; the former is located at the first maximum of the time evolution and the latter at the first maximum of the enveloping curve. This corresponds to the shape or a general sum (or product) of cosines, corroborating our previous mathematical explanation.

In general, the observation of both times is an easy task, but it is not guaranteed: as both times approximate in value, the interference between them increases, resulting in a larger shift of the first maximum, quickly deviating from the $T_{\mathrm{Cl}}$ analytical value, making the visualisation of TRe harder, if not impossible.

Summarising, there are two different ways to calculate regeneration times. The first one is looking for the maxima in the modulus of the autocorrelation function by numerically searching for the first maximum of the function (for $T_{\mathrm{Cl}}$) or the first maximum of its enveloping curve (for TRe). The second one is using the analytical expressions given in Equations (9) and (10).

#### 3.3.1. Classical Time

**Figure 6:**
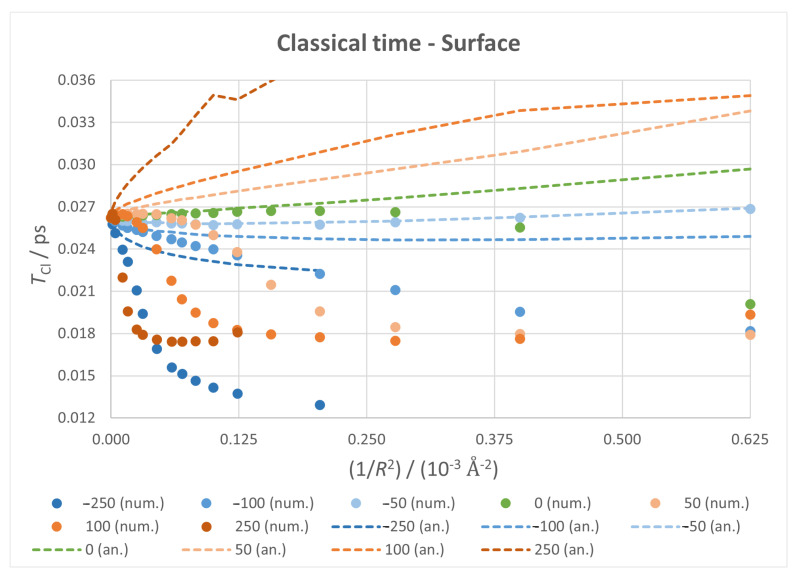
Classical time against $1/R^{2}$ for all fixed surface calculations, with markers for numerical values and dotted lines for analytical ones.

**Figure 7:**
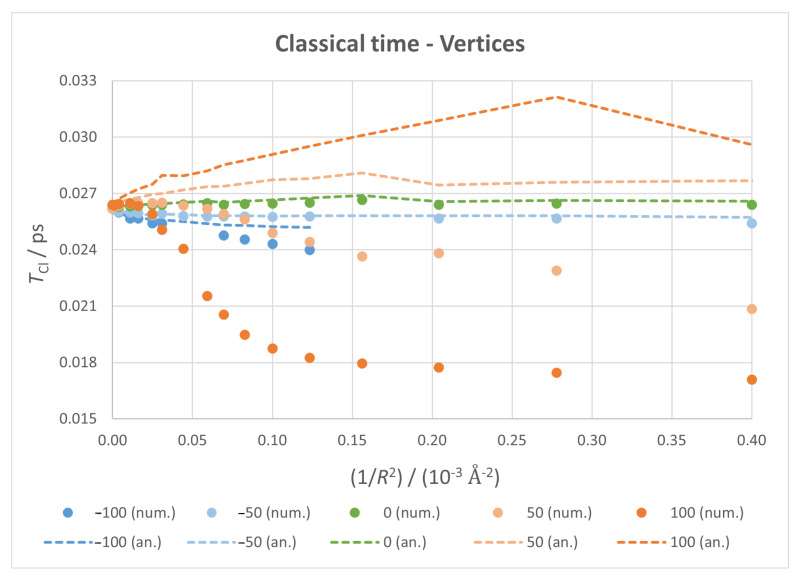
Classical time against $1/R^{2}$ for all fixed vertices calculations, with markers for numerical values and dotted lines for analytical ones.

Numerical and analytical values of classical time are plotted against $1/R^{2}$ in Figure 6 for the fixed surface case and in Figure 7 for the fixed vertices case. Taking the fixed surface as an ideal situation, we can see that, without any field applied, both numerical and analytical values of $T_{\mathrm{Cl}}$ grow with $1/R^{2}$ for low curvatures; this trend does not last, as a heavy deviation between them arises as we progress to more curved flakes, resulting in an overall monotonic growth for analytical $T_{\mathrm{Cl}}$ and a seemingly parabolic plot for numerical $T_{\mathrm{Cl}}$. The discrepancy between the two values is attributed to the interference from TRe. Analytical $T_{\mathrm{Cl}}$, being inversely related to the first derivative, shows a compression of the spectrum in the levels studied. The small distortion on the highest positive field is due, as will become clear later, to an important shift on the levels of the packet.

This base trend is intensified with positive fields, with the discrepancy happening at lower curvatures and lower values of $T_{\mathrm{Cl}}$ with stronger fields, reaching a limit value at a certain $1/R^{2}$. For negative fields, both values start with a decrease—with a good agreement between them—and seem to stabilise with an increasing curvature. These limit values barely change with the field intensity but do with its direction.

The fixed vertices case is similar, though there are sudden changes on the plot due to the shift to a more optimal structure as the curvature grows. However, the main effects are still observable, so previous analysis and conclusions still apply.

#### 3.3.2. Revival Time

**Figure 8:**
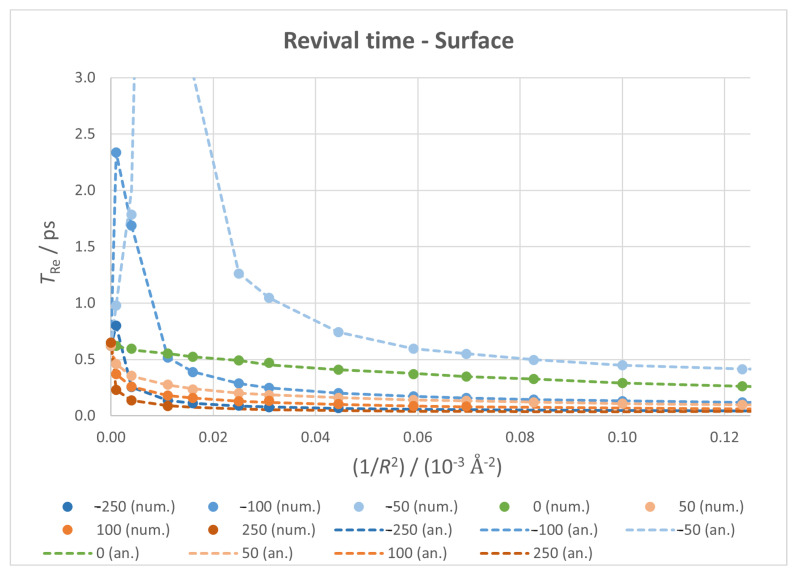
Revival time against $1/R^{2}$ for all fixed surface calculations, with markers for numerical values and dotted lines for analytical ones.

The results obtained for the revival time, which are depicted in Figure 8, showcase two very different behaviours according to the direction of the field. From the initial decrease at zero field conditions, positive fields make it reach lower values at a faster rate; negative fields cause a clear divergence of TRe, reaching a maximum—infinite, ideally—at a certain value of $1/R^{2}$, which shifts to a lower curvature as the field intensifies. In contrast to the classical time case, here both numerical and analytical values are very similar (indicating no interference from super-revival time).

**Figure 9:**
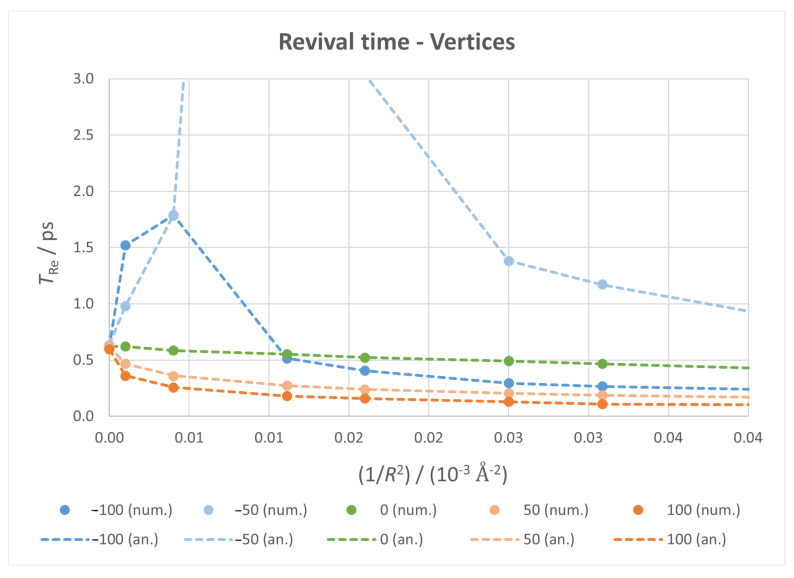
Revival time against $1/R^{2}$ for all fixed vertices calculations, with markers for numerical values and dotted lines for analytical ones.

As before, the fixed vertices case (Figure 9) merely introduces small jumps to this behaviour, but the general trends remain, which allows for an experimental setup to reach similar results to the ideal case.

By looking at the results presented in Figure 6, Figure 7, Figure 8 and Figure 9, it can be seen that both $T_{\mathrm{Cl}}$ and TRe lie in the 0.01–1 ps range (except for the divergences in revival times). Since typical molecular vibrational frequencies vary from less than $10^{13}$ Hz to approximately $10^{14}$ Hz, there could be some interference from atomic nuclei motions. Our calculations make use of the Born–Oppenheimer approximation and do not consider these motions. Nevertheless, taking into account that the mean amplitude of atomic vibrations in graphite at low temperatures is approximately 0.05 Å [86,87] and that the diagonal of our nanoflake is 46.6 Å their effects can be neglected.

#### 3.3.3. Electronic Spectrum

**Figure 10:**
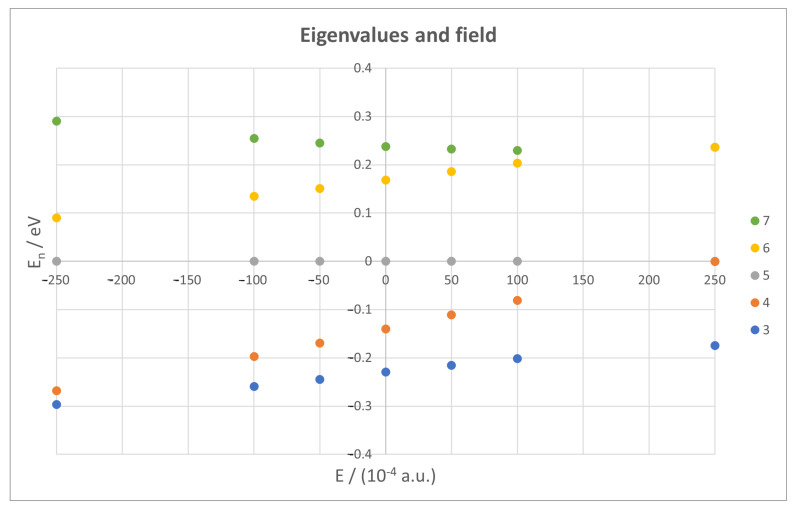
Eigenvalues for all five levels contributing to the wavepacket against external field, for a nanoflake with R=100 Å.

The origin of all observed features for both regeneration times lies within the electronic spectrum. Figure 10 shows how the five levels of the wavepacket change with the external field, taking the central one as the energy origin. A clear effect of the field is the shift of upper and lower levels, reaching an almost perfect degeneracy of n=5 with n=4 for big positive fields and a near degeneracy with n=6 for negative ones. While all eigenvalues change with the field, as expected, the ones of interest experiment a greater effect, which ultimately translates to the regeneration times observed.

**Figure 11:**
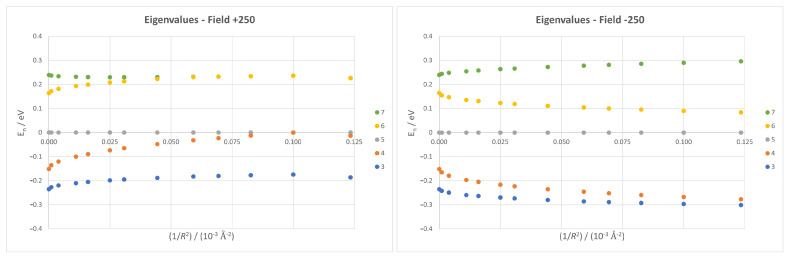
Eigenvalues for all five levels at fields +250 (**left**) and −250 (**right**) plotted against Gaussian curvature. All data correspond to fixed surface nanoflakes.

Analysing the spectrum at a fixed field value yields similar results, although there are clear differences depending on the field direction, as can be seen in Figure 11. Here, we can also appreciate further effects of both the field and curvature, like the opposite effect of the curvature increase on positive or negative fields, and even some crossings between levels, explaining the distortions on $T_{\mathrm{Cl}}$ at high positive fields.

The main consequence is the appearance of the divergence for negative fields. Taking a full view at the spectrum we can deduce that, since it changes from a degeneracy with the upper level to another with the lower one, there is one field value that makes both energy differences equal and thus the spectrum symmetric in this range. From an analytical point of view, and recalling Equations (13) and (14), this would mean a=b, leading to the following results:

(21) $$T_{\mathrm{Cl}}=\frac{2\pi \hbar }{a}\;\mathrm{and}$$

(22) TRe=2πħ0/2!=∞,

giving an infinite TRe, explaining the divergence, and a finite $T_{\mathrm{Cl}}$ value. From a numerical perspective, this results in the following:

(23) $${\left|A\left(t\right)\right|}^{2}\approx 4|c_{4}{|}^{2}|c_{5}{|}^{2}\cos\left(\frac{a}{\hbar }t\right)\cos\left(\frac{0}{2\hbar }t\right)=4|c_{4}{|}^{2}{\left|c_{5}\right|}^{2}\cos\left(\frac{a}{\hbar }t\right)\;,$$

with a single wave of a 2πħ/a period modulated by another one of an infinite period, matching the analytical values.

The same analysis can be performed with the extreme cases, on which the central level becomes degenerate with the upper or lower one, resulting in *a* or *b* becoming null. For example, for positive fields (a=0),

$$E^{\prime }\left(n_{0}\right)=bn_{0}+\frac{1}{2}\left(b-2bn_{0}\right)=\frac{b}{2}\;,$$

(24) $$T_{\mathrm{Cl}}=\frac{2\pi \hbar }{b/2}=\frac{4\pi \hbar }{b}\;,$$

$$E^{\prime \prime }\left(n_{0}\right)=b\;,$$

(25) TRe=2πħb/2!=4πħb.

Both classical and revival times would reach an equal value of double the classical time corresponding to *b*, as can be seen on the temporal evolution, again presenting a single frequency. This time, however, it is truly a single oscillation:

(26) $$\begin{array}{c} {\left|A\left(t\right)\right|}^{2}\approx 4|c_{4}{|}^{2}{\left|c_{5}\right|}^{2}cos\left(\frac{b}{2\hbar }t\right)cos\left(\frac{-b}{2\hbar }t\right)=\\ =4|c_{4}{|}^{2}{\left|c_{5}\right|}^{2}cos^{2}\left(\frac{b}{2\hbar }t\right)\;, \end{array}$$

resulting in both periods having the same value of 4πħ/b and a final temporal evolution comprised of a single wave of *half* the frequency, which is a squared cosine, as observed. The finite value of TRe is, of course, impossible to observe on the autocorrelation plot due to its lack of modulated superstructure. The main conclusion about this analysis is that the analytical approach is consistent with the numerical value for a narrow enough wavepacket, as long as both half-sum and half-difference—of the two main energy differences, that is—are not close in value.

Finally, all levels contributing to the wavepacket have been studied as a whole in order to check if under certain conditions of field or curvature the spectrum presented a regular distribution, similar to a harmonic oscillator, via the standard deviation of the energy differences between consecutive levels. The divergences observed were a strong hint, although they only account for the central levels. No combination of field and curvature shows any global regularity spanning all five levels, and the most equally spaced case is not regular enough to represent a truly harmonic oscillator.

## 4. Conclusions

This paper about curved graphene quantum dots expands our previous results by applying an external, perpendicular electric field to a spherical curved nanoflake, in order to complement the results obtained for different Gaussian curvatures without a field.

From a mechanical point of view, positive—towards the deformation—fields yield a curved nanoflake as the most stable configuration, as observed experimentally, while negative—opposing the deformation—fields cause the destabilisation of the system, which is capable of overcoming the bending effect for strong enough fields. Minimally restricted systems achieve further stabilisation, while the general trends remain unchanged. The electric dipole moment shows a clearly linear dependence with the field value, including flipping its direction as the field does the same; its magnitude increases as the deformation grows, with a small balance between opposite phenomena for negative fields.

As for the regeneration times, we have observed a practical equivalency between the same curvature and field. Classical times show initially opposite tendencies but an overall decrease towards a constant value, which is different depending on the field direction. Revival times show a monotonous decrease for positive fields and a divergence for certain values of $1/R^{2}$ for negative fields, similar to the one previously obtained for the hyperbolic case and also others present in graphene and silicene systems in an external magnetic or electric field, respectively. The connection between our results and these similar looking phenomena, as well as the observation of pseudo-magnetic fields on strained graphene, is yet to be fully justified. Lowering the nanoflake fixation to the minimum—vertices only—mildly distorts these results.

Finally, the analysis of the electronic spectrum provides an explanation for all regeneration times trends: the external field causes an energy shift of the levels in the wavepacket, with the central one reaching degeneracy with the upper or lower level depending on the field intensity and direction. Near-degenerate situations at intense fields give constant, equal classical and revival times; a symmetric level distribution gives rise to the observed divergences. This fine control in regeneration times achieved by adjusting the field and curvature could allow for the study of new phenomena on graphene. For instance, as we pointed out in the Introduction, it has been recently shown that double quantum dots can be created by the pseudo-magnetic fields in nanobubbles and that their quantum states can be manipulated to create a controllable qubit. By changing the curvature and the intensity of the electric field, the properties of these qubits could be optimised.

## Data Availability

The data presented in this study are contained within the article.

## Abbreviations

The following abbreviations are used in this manuscript:

DFT	Density Functional Theory
GGA	Generalised Gradient Approximation
LDA	Local Density Approximation
LUMO	Lower Unoccupied Molecular Orbital
